# The Use of Compressed Air in Dentistry

**Published:** 1896-01

**Authors:** S. Freeman

**Affiliations:** New York


### The Use of Compressed Air in Dentistry.

BY S. FREEMAN, NEW YORK.

Abstract of a paper read at the American Dental Association, August, 1895.

The dentist, who is called upon to treat and relieve the pain of such delicate and sensitive structures as the teeth, which bear an intimate anatomical and physiological relation to some of the most important organs of the body, should naturally look forward to that which can assist him in the practice of his profession. It is therefore essential to his success that he be provided with all necessary appliances which will aid him in his operations and mitigate the sufferings of his patients.

With this aim in view, no procedure appears to me to be more conducive to success than the application of compressed air, an agent which I employ extensively in operative dentistry, and it affords me great pleasure to call your attention to the divers instruments which are in use, and the application of which I will explain and demonstrate in detail.

Permit me to explain the methods of producing compressed air and the manner of conducting the same to your operating chair. It is obtained by means of a suction pump which sucks in the air and forces it through a pipe to a reservoir, as it is necessary for our purpose to have a continuous and considerable current. I would not advise the use of a hand pump or small cylinder, as in employing them you will find that both the air and the operator become quickly exhausted. I would, therefore, recommend that which is known as the "champion beer pump" or the compound pump of the same manufacture, for I find them both the simplest in construction as well as the most satisfactory air compressors on the market to-day.

Before placing the pump in your office, ascertain how many pounds of water pressure you have; if, as in my office, you have only twenty pounds the champion pump will not furnish, contrary to the claim of the manufacturers, the same amount of air pressure as water; as there is, as I have discovered, a loss of a few pounds; such being the case, the compound pump (although a few pounds of pressure are also lost) is preferable, as it is frequently necessary to have the air at a pressure of forty to fifty pounds to the square inch.

This pump is connected with the reservoir, which is a tank containing eighteen gallons of air tested to one hundred and fifty pounds pressure to the square inch: from this runs a quarter of an inch block-tin pipe, with branches to my laboratory and operating room; to this pipe I have attached a regulator and a gauge, the regulator being nothing more than a screw valve, as you loosen the screw you close the valve, and vice versa. From the regulator a pipe leads to the gauge, which indicates the number of pounds pressure. I use the one hundred pounds gauge, as it enables me to ascertain the maximum amount of pressure in my office. The pressure generated by the compound pump is sometimes so great that a lower gauge will not register it. To the gauge is connected a distributing pipe with three small cocks to attach the rubber tubes and the cut-off. The tubes should be of heavy rubber so as to withstand a high air pressure. The cut-offs close automatically.

In the practice of dentistry it is absolutely necessary to have the mouth in an antiseptic condition; to produce this effect no doubt many of you use the hand atomizer, which, by the continuous pressure of the bulb, causes a slight cramp in the hand; with the compressed air apparatus we do away with this work, you have the tube steadied, and can thoroughly cleanse the mouth or any cavity. I derive excellent results from the following prescription:—

| | | | |
|---|---|---|---|
| ℞ | Borine | - - - - - - - | 1 part. |
| | Pyrozone Med. 3 percent. | - - | 2 " |
| | Water | - - - - - - - | 1 " |

This makes an agreeable mouth-wash, applied with a spray.

No doubt you have frequently met cases where you would prefer to place on the rubber dam, but owing to the patient's inability to breath through the nostrils, you were compelled to send your patient away or otherwise work at a great disadvantage.

How often have you cast about for a remedy? It is a simple one; a two percent solution of cocain placed in an atomizer attached to your compressed air apparatus, which is gauged at ten to fifteen pounds pressure, and the spray thrown into the nostrils will invariably relieve that posterior nasal catarrh or reduce the swollen tissues of the nares to such an extent that you can proceed with your work in a few minutes without any inconveniences to your patient. In using the spray for this purpose have your patient sitting upright in the chair, the head inclined slightly forward. Insert the tube horizontally into the nares and do not apply over ten to fifteen pounds of air pressure, as otherwise you may set up an irritation of the middle ear. In stomatitis of the various kinds, the spray employed with a high pressure produces excellent results. In apthous stomatitis, which usually requires about a week to ten days to heal, I have succeeded in obtaining a cure in two days. Probably the citation of the following cases will enable you to [illegible]derstand the value of this agent.

Mr. H., age se[illegible]enteen years, applied to me for treatment May 7th, 1895, suffering from large grayish patches situated on the lower and upper lips. These patches were very painful; I applied the following prescription:

℞ Pyrozone Med. 3 percent. - - ℥iv.
Borine - - - - - - - - ℥ii.

with my atomizer under forty pounds pressure; in about two minutes it produced bleeding of the sores, washing away the grayish patch, and you could see the ebulition of the pyrozone, leaving escharotic spots similar to those produced by the application of the caustic pyrozone. I gave the patient the mouth-wash recommended by Dr. Sudduth, of pyrozone and soda mint; the patient reported on the ninth day of May having no sign of the patches. This method of treatment was not painful and proved very efficient, as I have used it in similar cases with uniformly good results.

A patient affected with mercurial stomatitis presented himself to me on June 5th, 1895. I found that characteristic lesion of the edges of the gums with considerable ptyalism, which excoriated the corners of the mouth, and was informed by the patient's physician that some time previous to the appearance of this disease, the patient had been taking mercury, three times a day, for three months, and during this medication, he would discontinue the use of the drug as soon as the teeth were sore to the touch. He was having his teeth attended to by a fellow-practitioner, who having learned his condition, upon the appearance of this lesion, treated the parts locally with glycerole of tannin and iodin, using, as a mouth-wash, Listerine. After treating the patient for three weeks without success, he concluded that it was secondary syphilis, and sent the patient to his physician for treatment. The physician, recognizing the condition as a mercurial stomatitis, referred the patient to me.

I used the pyrozone med. 3 percent on the diseased parts with a spray gauged at thirty pounds pressure, and gave him for a mouth-wash: Pyrozone Med. 3 percent, and Borine, equal parts.

When the patient applied to me, he could not eat his food without pain; the next day he reported, stating that it was the first time in some weeks that he enjoyed eating a meal. After three treatments with the pressure increased to fifty pounds, I found a decided improvement in the condition of the mouth.

The sloughing of the edges of the gum disappeared after the first treatment, and in four days the gums were in quite a healthy condition. At this time the patient was called away from the city, and I have since learned that the tissues are in a perfectly healthy condition. Before applying any medicines to the gums, it is always necessary to have a dry surface, so that the medicament may be readily absorbed and not distribute itself over the surface of the tissue.

I found it somewhat difficult to obtain a dry condition of the gums in the posterior portion of the mouth before employing this apparatus. Now I find it a very simple matter. By drawing back the corners of the mouth with a napkin or piece of cottonoid, and throwing the compressed air directly to the spot, it requires only a few seconds to procure the desired condition of the mucose, and upon applying your medicines, you get immediate absorption.

Now, I again have recourse to the air pressure, which seems to drive the medicines deep into the tissues.

In periostitis I prefer to use the cold-air current, which in itself gives relief to the patient.

In this manner the application of counter-irritants, sedatives, and local anæsthetics is made easy. With cocain you do not obtain as deep an anæsthesia as you do by the hypodermic injection of the drug; with a four percent solution I was enabled to leave abcesses within twenty to thirty seconds without pain, and I have produced with a ten percent solution sufficient anæsthesia to extract teeth, and did not observe the toxic symptoms which you frequently have following an injection.

In the treatment of pyorrhea alveolaris, it is absolutely essential that every particle of deposit of whatever form shall be removed, and that the debris shall be thoroughly washed away, leaving no particles to be ulcerated out; to do this it requires a vigorous stream of water.

Dr. J. N. Farrar introduced for this purpose a set of four syringes, with delicate points. I use this bottle, attached to my compressed air apparatus at fifty to sixty pounds pressure, which throws a stream with such a force as to entirely cleanse the parts; then I use the Davidson spray tubes, filled with Pyrozone Med. 3 percent and Borine, equal parts' which throws out such copious spray that it lifts the gum tissues away from the teeth, introducing the medicament directly to the seat of trouble, forcing away all foreign particles and giving a clean antiseptic condition of the parts.

It has been demonstrated that when the water is removed from a tooth, the normal function of transmitting impressions seems to be modified. This desiccation can be accomplished by several agents, heat, cold and chemicals. We know that heat or cold will produce pain, and in the application of either we should proceed with extreme caution.

I use a hot-air syringe similar to the S. S. White No. 30, only it has twice as large a cylinder. The chamber is filled with carbon, which is found to be one of the best materials for retaining caloric, and only requires a few minutes over a Bunsen burner flame to accomplish the requisite amount of heat. With these syringes attached to the compressed air apparatus, you can so regulate the flow of air that in from one to five minutes you have your tooth thoroughly dry; then introduce your medicine heated to about 95°F., and then applying your warm-air current with about forty pounds pressure, you will be able to excavate your tooth without pain, nor will you have any subsequent irritation. With this method it is not necessary to employ acids in introducing your cocain, nor is much of your valuable time wasted waiting for the absorption of the medicament. You can also spray your medicines into a tooth, but I would first advise the dehydration of the dentine.

In the bleaching of teeth I find that by the application of hot air at a high pressure, I am able to produce the required condition in one-half the usual time, as you rapidly evaporate the pyrozone 25 percent and force it into the tubuli.

It is necessary to be cautious in bleaching teeth so as not to get them too white, as you will frequently observe them somewhat lighter colored on the following day.

In making a tooth perfectly aseptic, the same method of applying your drug as I have recommended in inducing anæsthesia, will give you a reliable antiseptic condition of both tooth and root.

In setting in pivot teeth, we often find it very difficult to carry the cement to the upper portion of the root.

In forcing it into the root canal with a dry instrument, upon withdrawal of the latter the cement is found adherent to it and not to the walls of the root; but with a high pressure you can force it into every irregularity of the root, while at the same time the compressed air will dry all the surrounding tissue and you avoid the necessity of wiping away the moisture excreted by the mucous glands.

